# *Weizmannia coagulans* BCF-01: a novel gastrogenic probiotic for *Helicobacter pylori* infection control

**DOI:** 10.1080/19490976.2024.2313770

**Published:** 2024-02-09

**Authors:** Zhenhui Chen, Ziyu Tang, Wendan Li, Xiaoshi Deng, Lu Yu, Jixiang Yang, Jiaxin Liu, Yunshui Cheng, Wanwen Huang, Xiaotong Guo, Jiamin Shan, Daixuan Zhou, Weisen Zeng, Yang Bai, Hongying Fan

**Affiliations:** a Department of Microbiology, Guangdong Provincial Key Laboratory of Tropical, Disease Research, School of Public Health, Southern Medical University, Guangzhou, China; b Department of Radiation Oncology, Nanfang Hospital, Southern Medical University, Guangzhou, China; c Department of Traditional Chinese Medicine, Nanfang Hospital, Southern Medical University, Guangzhou, China; d Guangdong Provincial Key Laboratory of Gastroenterology, Inst. of Gastroenterology of Guangdong Province, Department of Gastroenterology, Nanfang Hospital, Southern Medical University, Guangzhou, China; e Department of Cell Biology, School of Basic Medicine, Southern Medical University, Guangzhou, China

**Keywords:** Human gastrogenic probiotic, *weizmannia coagulans* BCF-01, *helicobacter pylori* infection, gastric microbiota homeostasis, mucosal immunity

## Abstract

The widespread prevalence of *Helicobacter pylori* infection, particularly in China, contributes to the development of gastrointestinal diseases. Antibiotics have limitations, including adverse reactions and increased antibiotic resistance. Therefore, identification of novel gastrogenic probiotics capable of surviving the acidic gastric environment and effectively combating *H. pylori* infection has potential in restoring gastric microbiota homeostasis. Five novel strains of human gastrogenic *Weizmannia coagulans* (BCF-01–05) were isolated from healthy gastric mucosa and characterized using 16S rDNA identification. Acid resistance, *H. pylori* inhibition, and adherence to gastric epithelial cells were evaluated in *in-vitro* experiments and the molecular mechanism explored in *in-vivo* experiments. Among the gastric-derived *W. coagulans* strains, BCF-01 exhibited the strongest adhesion and *H. pylori* inhibition, warranting further *in-vivo* safety evaluation. Through 16S rRNA sequencing of a mouse model, BCF-01 was determined to significantly restore *H. pylori*-associated gastric dysbiosis and increase the abundance of potential probiotic bacteria. Furthermore, BCF-01 enhanced mucosal tight junction protein expression and inhibited the TLR4-NFκB-pyroptosis signaling pathway in macrophages, as demonstrated by qRT-PCR and western blotting.

These findings highlight the potential of BCF-01 in the prevention and control of *H. pylori* infection. Specifically, treatment with BCF-01 effectively restored gastric microecology and improved *H. pylori*-mediated mucosal barrier destruction while reducing inflammation through inhibition of the TLR4-NFκB-pyroptosis signaling pathway in macrophages. BCF-01 is a promising alternative to traditional triple therapy for *H. pylori* infections, offering minimal side effects with high suitability for high-risk individuals.

## Introduction

*Helicobacter pylori*, the primary pathogenic bacterium in the stomach and a human carcinogen, is closely associated with gastrointestinal diseases such as chronic gastritis.^1,2^ Approximately 50% of the global population is infected with *H. pylori*, with China being one of the most heavily affected countries.^3^ *H. pylori* infection disrupts the stomach microbiome, leading to a significant reduction in colonization by various probiotics, thereby causing gastric dysbiosis.^4,5^ Current guidelines recommend classic bismuth quadruple therapy (proton pump inhibitors, bismuth, and two antibiotics) for *H. pylori* infections.^6,7^ However, antibiotic use often results in adverse reactions, including diarrhea, which reduce treatment compliance and contribute to the global increase in *H. pylori* resistance.^8,9^

In recent years, probiotics have emerged as promising candidates for preventing and treating *H. pylori* infection via restoration of gastric microbiota homeostasis and limiting adverse reactions.^10–13^ However, classic probiotic treatment often exhibits a low *H. pylori* eradication rate and inconsistent effectiveness.^14,15^ This may be attributed to the unsuitability of gut-derived probiotics in colonizing the unique environment of the stomach. Therefore, it is crucial to isolate and identify gastric-derived probiotics capable of tolerating acidic gastric environments and effectively treating *H. pylori* infections.

Previously, 16S rRNA sequencing and bioinformatic analysis revealed a significant decrease in the abundance of the *Bacillus* and *Lactobacillus* genera in *H. pylori*-infected patients.^16^ *Weizmannia coagulans*, a spore-forming probiotic, has garnered considerable research attention owing to its probiotic properties and ability to withstand extreme environmental conditions.^17,18^ Gut-derived *W. coagulans* has been reported to modulate immunity and restore intestinal microbiome homeostasis.^19,20^ However, research surrounding gastric-derived *W. coagulans* is limited.

Therefore, we aimed to isolate gastric-derived probiotics from the human gastric mucosa that can effectively and safely inhibit *H. pylori* infection. We also investigated the molecular mechanisms underlying the prevention and treatment of *H. pylori* infection by these probiotics, thereby providing a foundation for developing an effective treatment strategy to combat *H. pylori* and restore gastric microbiota homeostasis.

## Materials and methods

### Bacterial strains, cells, and mouse models

*H. pylori* (strains SS1, ATCC 26,695, and ATCC 43,504) were provided by the Department of Gastroenterology, Nanfang Hospital. *W. coagulans* (strain ATCC7050), *Lactobacillus rhamnosus* (strain ATCC53103), GES-1 cells (human gastric mucosal epithelial cells), and RAW264.7 (mouse monocyte macrophage leukemia cells) were obtained from the American Type Culture Collection. *H. pylori* was cultured at 37°C in Columbia blood agar plates (Guangdong Huankai Microbial Sci. & Tech., Guangzhou, China). *W. coagulans* was grown at 45°C in improved de Man Rogosa Sharpe (MRS) liquid medium (Guangzhou Huaikai Microbial Sci.& Tech.). *L. rhamnosus* was cultured at 37°C in MRS medium. GES-1 and RAW 264.7 cells were grown in Roswell Park Memorial Institute-1640 (RPMI-1640; Gibco, USA) with 10% fetal bovine serum (FBS; Gibco).

All animal experiments were approved by the Institutional Animal Care and Use Committee of Nanfang Hospital. We obtained four-week-old male and female C57/BL6 mice from the Southern Medical University Laboratory Animal Center, China, and housed them under specific pathogen-free conditions. All *in-vivo* experiments were performed in accordance with our institution’s guidelines.

### Clinical specimen collection, bacterial isolation, and purification

Gastric antral biopsy samples were collected from the Department of Gastroenterology at Nanfang Hospital, Southern Medical University. The Ethics Committee of Nanfang Hospital approved the research protocol (NFEC-2022-252).

Biopsy specimens were immediately placed in centrifuge tubes containing brain-heart infusion liquid medium (Guangzhou Huankai Microbial Sci. & Tech.), and transported to the laboratory at 4°C. Gastric mucosal tissues were homogenized until no large tissue fragments were visible. The resulting suspension was added to MRS liquid medium and incubated at 45°C in a DG250 Anaerobic Culture Chamber (Don Whitley Scientific, UK) for 24–48 h. After pre-culturing, the bacterial suspension was streaked onto MRS solid agar plates and incubated for an additional 24–48 h. Once single colonies had grown, colonies were isolated via streaking. Isolated colonies were assessed for color, size, and edge morphology; colonies that met the desired criteria from *Bergey’s Manual of Determinative Bacteriology* were selected for further cultivation and preserved at −80°C in 30% glycerol.

W. *coagulans* (strain BCF-01) was preserved at Guangdong Microbial Culture Collection Center (preservation number: GDMCC No. 62386).

### Gram staining

The preserved BCF-01–05 bacteria were revived by culturing in MRS liquid medium at 45°C for 24 h. Bacteria was spread onto a sterilized glass slide with 10 μL of PBS. The slides were fixed before staining with crystal violet for 1 min, followed by rinsing with water. Gram’s iodine solution was added to the slides for 1 min before rinsing with water. Next, slides were washed with 95% ethanol for 20 s before rinsing with water. Safranin staining was performed for 1 min followed by rinsing with water. Excess water was removed, and the slides were air-dried. The bacteria were observed under a microscope (Olympus, Japan) at 100× magnification to assess their size, morphology, arrangement, and Gram-staining characteristics.

### Matrix-assisted laser desorption/ionization time of flight (MALDI-TOF) analysis

For MALDI-TOF analysis, 1 μL of matrix was added to the target plate (Bruker, USA). The BCF-01–05 suspension was spread onto a small hole in the target plate and mixed with the matrix before air-drying. The target plate was inserted into the loading slot; here, a laser ionized the bacterial components, vaporizing the sample molecules, and transferring them into a vacuum. Charged particles were accelerated with a high voltage before entering a time-of-flight mass analyzer. After detecting the mass charge ratio of each ion, the peptide mass spectra were generated for each peptide segment, representing the peptide fingerprint. Species identification was conducted by comparing peptide mass spectra with those in the Biotyper bacterial database.

### Strain identification

Bacterial genomic DNA was extracted using a genomic DNA extraction kit (Qiagen, Hilden, Germany) according to the manufacturer’s instructions. The isolated 16S rDNA fragments were amplified via PCR using universal primers F27 (5′-AGAGTTTGATCCTGGCTCAG-3′) and R1492 (5′-GGTTACCTTGTTACGACTT-3′) and a thermocycler (Agilent SureCycler 8800, USA). The PCR reaction mixture included 2 μL of template DNA, 10 μL of Premix Taq DNA Polymerase (Takara Bio, Shiga, Japan), 1 μL of each primer, and 6 μL of nuclease-free water. The PCR program comprised: initial denaturation at 94°C for 5 min; 30 cycles at 94°C for 60 s, 60°C for 60 s, and 72°C for 90 s; and final extension at 72°C for 10 min. PCR products were visualized on an ethidium bromide – stained agarose gel. The 16S rDNA sequences were analyzed using BLAST (http://www.ncbi.nlm.nih.gob/blast).

### Acidic pH and bile salt tolerance

To evaluate resistance to gastric acid conditions, bacterial cells were harvested from overnight cultures and placed in tubes at pH 2.0, 3.0, or 4.0 for 3 h. To determine resistance to simulated intestinal fluid, the isolated bacteria were cultured in MRS broth (Guangdong Huankai Microbial Sci. & Tech.) containing 0.3% bile salts (Solarbio, Beijing, China) and incubated at 37°C for 8 h. Bacterial growth was determined by measuring absorbance at 600 nm using a spectrophotometer (Annoron, China).

### Antimicrobial susceptibility

*H. pylori* bacteria were streaked onto Columbia blood agar plates. The BCF-01-05 and ATCC 7050 culture supernatants were collected, adjusting the OD_600_ to 1. Then, 1 mL of the bacterial suspension was centrifuged at 12,000 rpm for 2 min before the supernatant was harvested. This supernatant was filtered using a 0.22-μm filter membrane (Merck Millipore, USA); the bacterial pellet was washed three times and resuspended in PBS. Subsequently, the bacterial suspension, supernatant, and PBS-resuspended bacterial cells were added to an Oxford cup on double-layer blood agar plates; MRS liquid medium and PBS were added to the blank control groups. All plates were incubated at 37°C under microaerophilic conditions for 48 h. Then, the inhibition zone around each well was measured.

### Co-aggregation

Co-aggregation was performed with stationary-phase cells of *W. coagulans*, and *H. pylori* cells were stained separately using either hexidium iodide (HI, 10 μg/mL) or carboxyfluorescein diacetate succinimidylester (CFDA-SE, 1 μg/mL, MedChemExpress, USA) according to the manufacturer’s instructions. Excess dye was removed by extensive washing with PBS. Equal amounts of cells were mixed and vortexed for 10 s prior to phase-contrast and fluorescence microscopy. For some experiments, cells were mixed, and immediate flocculation was observed. Co-aggregates could be observed visually as flocking structures, whereas no such structures were present in the controls of the single strains. If no aggregates were detected after 10 min, pairs were judged as non-coaggregating.^21^

### RNA isolation and qRT-PCR

Total RNA was extracted from the gastric contents (0.1 g) using TRIzol reagent (Takara Bio). Thereafter, the extracted RNA was converted to cDNA using a reverse transcription kit (Takara Bio). Gene expression was then determined using qPCR SYBR Green Master Mix (Takara Bio) and the 7500 real-time quantitative PCR system (Applied Biosystems, USA). The relative quantification of target gene expression levels was determined via the 2^−ΔΔCT^ method, using GAPDH and 16S for normalization. The primers used in this study are listed in Table 1.

**Table 1.** Primers of qPCR.

Species	Gene symbol	Forward primer (5’-3’)	Reverse primer (5’-3’)
Bacteria	CagA	GTTGATAACGCTGTCGCTTCA	GGGTTGTATGATATTTTCCATAA
Bacteria	VacA	CACCCACTTGATTATTCACTCTTAAA	CCACCCAATAACACGCAAA
Bacteria	16S v3-v4	CCTACGGGNGGCWGCAG	GACTACHVGGGTATCTAATCC
Mouse	RORγ	GACCCACACCTCACAAATTGA	AGTAGGCCACATTACACTGCT
Mouse	Foxp3	ACCATTGGTTTACTCGCATGT	TCCACTCGCACAAAGCACTT
Mouse	IL-17α	TTTAACTCCCTTGGCGCAAAA	CTTTCCCTCCGCATTGACAC
Mouse	TGF-β	GCCTGAGTGGCTGTCTTTTG	AGTTGGTATCCAGGGCTCTCC
Mouse	IL-10	CTTACTGACTGGCATGAGGATCA	GCAGCTCTAGGAGCATGTGG
Mouse	Arg-1	TGTCCCTAATGACAGCTCCTT	GCATCCACCCAAATGACACAT
Mouse	IL-1β	GAAGTTGACGGACCCCAAAA	CCACAGCCACAATGAGTGATAC
Mouse	IFN-γ	ATGAACGCTACACACTGCATC	CCATCCTTTTGCCAGTTCCTC
Mouse	IL-4	GGTCTCAACCCCCAGCTAGT	GCCGATGATCTCTCTCAAGTGAT
Mouse	TNF-α	CCTGTAGCCCACGTCGTAG	GGGAGTAGACAAGGTACAACCC
Mouse	IL-12	CAATCACGCTACCTCCTCTTTT	CAGCAGTGCAGGAATAATGTTTC
Mouse	IL-6	AGTCCTTCCTACCCCAATTTCC	GTCTTGGTCCTTAGCCACTCC
Mouse	GAPDH	AGCTTGTCATCAACGGGAAG	TTTGATGTTAGTGGGGTCTCG
Human	IL-6	CACTGGTCTTTTGGAGTTTGAG	GGACTTTTGTACTCATCTGCAC
Human	IL-1α	CTTCTGGGAAACTCACGGCA	AGCACACCCAGTAGTCTTGC
Human	IL-1β	TGAGCTCGCCAGTGAAATGA	AGATTCGTAGCTGGATGCCG
Human	TGF-β	GGCCAGATCCTGTCCAAGC	GTGGGTTTCCACCATTAGCAC
Human	ZO-1	CAACATACAGTGACGCTTCACA	CAACATACAGTGACGCTTCACA
Human	GAPDH	GGGAGCCAAAAGGGTCATCA	TGATGGCATGGACTGTGGTC

### H. pylori cell adhesion assay

GES-1 cells were cultured in 96-well plates (Corning, NY, USA) until cell density reached approximately 80% confluence. The culture medium was replaced with 50 μL of RPMI-1640 containing 10% FBS. This experiment consisted of four treatment groups: a control group, which received 50 μL of RPMI-1640; a *H. pylori* group, which received 50 μL of RPMI-1640 containing *H. pylori* at a multiplicity of infection (MOI) of 1:100; a preventive group, which received 25 μL of RPMI-1640 containing *W. coagulans* at an MOI of 1:1000, followed by 25 μL of RPMI-1640 containing *H. pylori* after 2 h; and a competition group, which received 25 μL each of RPMI-1640 containing *H. pylori* and *W. coagulans* at MOIs of 1:100 and 1:1000, respectively. After incubation at 37°C in a 5% CO_2_ incubator for 2 h, cells were washed three times with PBS. Then, 100 μL of urease detection reagent was added to each well, before measuring absorbance at 540 nm. The relative adherence rate of *H. pylori* (%) was calculated using the following formula: (Experimental group absorbance – Control group absorbance)/(*H. pylori* group absorbance – Control group absorbance) × 100.

Next, cell adhesion assays were conducted. GES-1 cells were prepared in confocal dishes, with the same incubation and grouping methods as those described above. *W. coagulans* and *H. pylori* were labeled with fluorescein isothiocyanate and Texas Red dye (Sigma-Aldrich, USA), respectively, at a concentration of 1:100. After co-incubation at 37°C and 80 rpm for 12 h, the supernatant was removed, and the cells were washed three times with PBS. Fluorescence intensity was observed using a fluorescence microscope Ti-s (Nikon, Tokyo, Japan); images were captured for analysis.

### In-vivo colonization

Stock and working solutions were prepared according to the experimental protocols. Click A stock solution was prepared by adding 500 μL of DMSO (Sigma-Aldrich) to the original Click-iTTM ManNAz metabolic glycoprotein labeling reagent (Thermo Fisher Scientific, MA, USA). Click B stock solution was prepared by adding 100 μL of DMSO to the original Click B dye (Click-iTTM sDIBO alkyne for copper-free click chemistry; Thermo Fisher Scientific). Then, 50 μM Click A working solution and Click B stock solutions were prepared in DMSO. *W. coagulans* was cultured to an OD_600_ of 1; then, 200 μL of Click A was added before incubation at 37°C in the dark for 24 h. The labeled bacterial cells were washed once and resuspended in PBS. Then, 5 μL of Click B was added before the mixture was incubated at 37°C in the dark for 1 h. The centrifuged bacterial pellet was resuspended in 200 μL of PBS.

The labeled bacteria were orally administered to C57/BL6 mice, and fluorescence signals were detected using a multimodal small-animal *in-vivo* imaging system (FX Pro, Bruker) 72 h after administration. Finally, mice were euthanized and the gastrointestinal tract was collected for further fluorescence imaging.

### Acute toxicity experiment

Animal studies were approved by the Institutional Animal Care and Use Committee of Southern Medical University. The male and female C57/BL6 mice were divided into two groups: control and *W. coagulans* groups. The control group was administered 200 μL of PBS via oral gavage; the *W. coagulans* group was administered 200 μL of *W. coagulans* (5 × 10^11^ CFU/mL) suspended in PBS via oral gavage. Following administration, the mice were continuously monitored for body weight, mental status, behavioral activity, mortality or moribund state, food intake, and fecal characteristics. At the end of the experiment, we collected cardiac blood from the mice and assessed their liver and kidney functions using a fully automated biochemistry analyzer (Mindray, China). Following euthanasia by cervical dislocation, the stomach, colon, small intestine, liver, kidney, and spleen were collected.

### Histology

All tissue samples were immediately fixed in 4% formalin and embedded in paraffin for histological analyses. After deparaffinization, sections (4 µm thick) were processed for hematoxylin and eosin (H&E) staining.

### H. pylori infection mouse model

Animal studies were approved by the Institutional Animal Care and Use Committee of Southern Medical University. Mice were randomly divided into five groups: Control, *H. pylori*, BCF-01, LGG+BCF-01, and triple therapy. In the Control group, mice were administered PBS by gastric gavage as a sham treatment. The *H. pylori* model group was constructed according to previous literature, by administering a combination of 10 mg/mL ampicillin, 2 mg/mL clarithromycin, and 25 mg/mL metronidazole (Solarbio) via gastric gavage daily for three days as pretreatment to disrupt the gastric flora and mucosal barrier; subsequently, these mice were infected by gastric gavage with 1 × 10^10^ CFU/ml of *H. pylori* suspended in 300 μL of PBS for 52 days. In the BCF-01 group, alongside the *H. pylori* model construction described above, mice received 1 × 10^10^ CFU/ml of BCF-01 (suspended in 300 μL of PBS) via gastric gavage 7 h after *H. pylori* infection for 7 days. The LGG+BCF-01 treatment was similar to that of the BCF-01 group; however, mice were administered 5 × 10^9^ CFU/ml of BCF-01 and *L. rhamnosus* (LGG). The triple therapy group involved *H. pylori* model construction alongside daily treatment with a combination of three antibiotics (2 mg/mL amoxicillin, 1 mg/mL clarithromycin, and 0.06 mg/mL omeprazole; Solarbio) with a total volume of 300 μL. Body weight changes were monitored every week. On day 60, the mice were euthanized, their stomach tissues were collected, and their gastric contents were used for subsequent experiments.

### Urease test and fecal H. pylori antigen detection

The urease test and fecal antigen detection were conducted according to the manufacturer’s instructions. For the urease test, we added 0.1 g of stomach tissue from the mice (ensuring consistent sampling location) to a 1.5-mL tube containing 200 μL of urease (Solarbio); color changes were observed for 20 min. For fecal antigen detection, 0.1 g of feces were collected from the mice and ground in 100 μL of PBS. This mixture was added to the wells of a colloidal gold test kit (Wondfo, Guangzhou, China); then, we observed the appearance of a positive strip following a 5-min incubation period.

### 16S rRNA sequencing of gastro contents

DNA was extracted using a DNA extraction kit (Tiangen, Beijing, China). Concentration and purity were measured using a NanoDrop One (Thermo Fisher Scientific). The V3–V4 region of the 16S rRNA gene was amplified using 338F and 806 R primers; samples were sequenced by Majorbio Bio-Pharm Biotechnology (Shanghai, China) using the Illumina HiSeq 2500 platform (Illumina, San Diego, CA, USA). Paired-end reads were merged via fast length adjustment of short reads, and sequence analysis was performed using UPARSE.^22^ Sequences with ≥ 97% similarity were grouped into the same operational taxonomic unit (OTU). The QIIME software was used to select representative sequences from each OTU, which were then compared and annotated according to the SILVA database (v138; https://www.arb silva.de/). The alpha diversity index of the bacterial communities was determined using usearch-alpha div (v10) in USEARCH (http://www.drive5.com/usearch/); the beta diversity index was determined using the R package vegan (v1.17) and the unweighted pair-group method with arithmetic mean. Finally, linear discriminant analysis effect size (LEfSe) analysis was performed using website ImageGP (http://www.ehbio.com/ImageGP/).^23^

### Protein extraction and western blotting

Protein samples were extracted from 0.5 g of gastro tissue using a protein extraction kit (Beyotime Biotechnology, China); these samples were then used for western blot analysis, which was performed according to the manufacturer’s instructions. A dilution ratio of 1:1000 was used for all the primary antibodies, including rabbit anti-NLPP3 polyclonal antibody (19771–1-AP, Proteintech), rabbit anti-TLR4 polyclonal antibody (A5258, Abclonal), rabbit anti-p65 monoclonal antibody (CST 8242T, CST, Danvers, MA, USA), rabbit anti-GSDMD monoclonal antibody (CST 69469S, CST), rabbit anti-Caspase 1 monoclonal antibody (CST 3866T, CST, Danvers), mouse anti-iκBα monoclonal antibody (CST 4814T, CST), rabbit anti-Myd88 polyclonal antibody (A0980, Abclonal), rabbit anti-IL-1β polyclonal antibody (A16288, Abclonal), rabbit anti-IL-18 polyclonal antibody (A1115, Abclonal), rabbit anti-ZO-1 monoclonal antibody (CST 13,663, CST), rabbit anti-Occludin monoclonal antibody (CST 91,131, CST), rabbit anti-RORγ polyclonal antibody (A10240 Abclonal), rabbit anti-Foxp3 monoclonal antibody (CST12632; CST), and mouse anti-β-actin monoclonal antibody (CST4970; CST). A dilution ratio of 1:2000 was used for all the secondary antibodies, including anti-rabbit IgG, HRP-linked antibody (CST7074, CST). Enhanced chemiluminescence was used for detection.

### Bone-marrow-derived macrophage isolation and cultivation

First, four-week-old C57/BL6 mice were euthanized via cervical dislocation and disinfected with 75% alcohol. The tibia and femur of the mice were isolated and extracted, ensuring that the bones remained intact and the surrounding soft tissues were removed. The tibia and femur were washed three times with PBS and transferred to a cell culture dish containing RPMI-1640 supplemented with 10% FBS. The ends of the tibia and femur were opened using ophthalmic scissors; then, a syringe containing 1 mL of RPMI-1640 was used to flush the bone marrow cells from one end of the bone. This was repeated several times until the bone turned white. Five times the volume of ACK red blood cell lysis buffer (LEAGENE, Beijing, China) was then added to the ruptured red blood cells. After centrifugation at 900 rpm, an appropriate amount of RPMI-1640 was added to resuspend the cells before filtering out impurities using a 100-μm cell filter (Nest, Wuxi, China). Centrifugation was conducted before supernatant removal; then RPMI-1640 with 10% FBS, double antibodies, and 10 ng/mL M-CSF (PERPROTECH, USA) was added to resuspend the cells and induce bone marrow cell differentiation into macrophages. Cells were counted using a hemocytometer and cell concentration was adjusted to 1 × 10^6^ cells/mL. Cells were incubated in a 5% CO_2_ incubator at 37°C, replacing the medium every two days. On day 7, primary bone-marrow-derived macrophages were obtained for use in further experiments.

### Statistical analysis

All data are expressed as mean ± standard deviation; statistical significance was set at *p* ≤ .05. Statistical differences between experimental groups were analyzed using the Student’s *t*-test using SPSS (v22.0; IBM, NY, USA). All experiments were performed in triplicates.

## Results

### Isolation, culturing, and functional characterization of gastric-derived W. coagulans

Previous sequencing results have demonstrated a decline in the abundance of bacterial abundance, including *Bacillus* and *Lactobacillus*, in gastric mucosal samples from patients with *H. pylori* infection.^16^ *Bacillus spp*., conventional probiotics, have shown beneficial effects in the gut. To explore the potential benefits and colonization ability of acid-resistant *Bacillus* strains, we isolated novel *Bacillus* strains from human gastric mucosal samples. Ultimately, five acid-resistant *W. coagulans* strains (BCF-01–05) with typical *Bacillus* characteristics were obtained (Figure S1).

**Figure 1.**
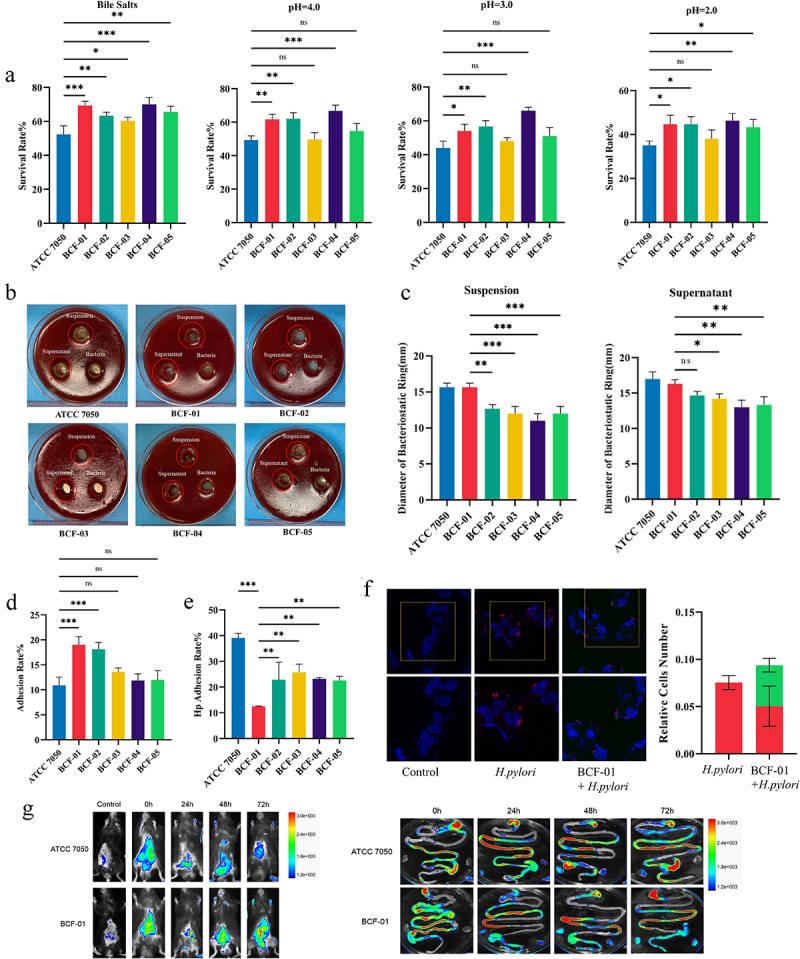
Characterization and colonization ability of gastrogenic *W. coagulans* strains.

To assess tolerance to acidic environments, acid resistance experiments were conducted on BCF-01–05 strains. These strains exhibited superior acid resistance compared to the reference strain, ATCC7050 (Figure 1a). At pH 2, the survival rate of ATCC7050 decreased to below 40%, whereas most gastric-derived strains maintained a survival rate > 40%. Additionally, some strains achieved survival rates of 60% at pH 4. Specifically, BCF-04 exhibited the highest acid resistance, followed by BCF-02 and BCF-01. These acid resistance profiles appeared strain specific. Nonetheless, all five strains exhibited significantly higher survival rates (>60%) than ATCC7050 (<60%) when exposed to simulated intestinal fluid (Figure 1a). However, BCF-01 and BCF-04 exhibited the highest acid tolerance under these conditions.

A. Survival rate of different *W. coagulans* strains in simulated intestinal fluid and simulated gastric fluid. B. Representative picture of anti bacteria test. C. Diameter of inhibition zones. D. Adhesion rate of different *W. coagulans* strains adhering to GES-1 cells using the plate coating method. E. Adhesion rate of Hp adhering to GES-1 cells. F. Representative fluorescent picture of Hp and BCF-01 adhering to GES-1 cells. G. Fluorescent intensity pictures of the *in-vivo* colonization experiment. *p < .05; **p < .01; ***p < .001.

The inhibitory effects of BCF-01–05 against *H. pylori* were determined using antibacterial tests. The strain suspensions and supernatants formed inhibition zones against *H. pylori*, whereas the bacteria itself did not. BCF-01 exhibited significantly stronger inhibitory effects of strains SS1, ATCC26695, and ATCC43504 than the other gastric-derived strains, with no significant difference to the performance of ATCC7050 (Figures 1b, c and S2). Furthermore, we conducted co-aggregation experiments and qPCR to explore the properties of *H. pylori* inside the BCF-01-05 subculture. The results of the co-aggregation experiments demonstrated that *H. pylori* exhibited the highest degree of co-aggregation with BCF-01 and BCF-02, suggesting a significant masking of the sites on *H. pylori* that adhere to epithelial cells (Figure S3A-B). The co-aggregation between *H. pylori* and BCF-01 appeared as noticeable flocking structures, which could be easily observed with the naked eye (Figure S3C). A qPCR was conducted to investigate the impact of gastric-derived strains on the pathogenic genes of *H. pylori*. The results indicate that BCF-01 can effectively suppress the transcription levels of CagA and VacA (Figure S3D).

We further assessed the effects of these strains on *H. pylori* adhesion to gastric cells (GES-1). BCF-01 exhibited enhanced adhesion to GES-1 cells compared to ATCC7050 and the other strains (Figures 1d and S4); moreover, BCF-01, in the preventive treatment group, significantly reduced *H. pylori* adhesion to GES-1 cells compared to all other strains. This *H. pylori* adhesion rate dropped to approximately 10% after BCF-01 pretreatment (Figure 1e). In the competition treatment group, BCF-01 remained the most effective at reducing *H. pylori* adhesion, although this difference was not significant (Figure 1f). *In-vivo* imaging confirmed that BCF-01 exhibited superior colonization in the stomach and intestines of mice, surpassing that of ATCC7050 (Figure 1g). After 72 h, ATCC7050 was nearly undetectable in the stomach of the mice, whereas BCF-01 exhibited strong fluorescence signals.

### BCF-01 safety

**Figure 2.**
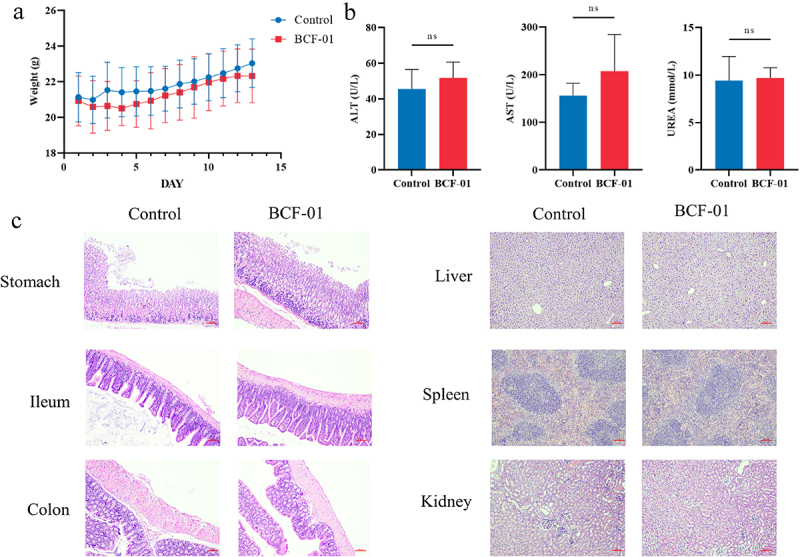
Safety evaluation of gastrogenic *W. coagulans BCF-01* in male mice.

Considering the superior performance of BCF-01, we conducted safety assessments to evaluate its potential for clinical use. Acute toxicity experiments in mice showed no significant differences in body weight between the BCF-01 and control groups (Figures 2a and S5A). ALT and AST blood levels in the BCF-01 group mice were not significantly different from those in the control group, indicating that BCF-01 did not exhibit significant hepatotoxicity (Figures 2b and S5B). UREA levels were also not significantly different from those of the control group, suggesting that BCF-01 did not exhibit significant nephrotoxicity (Figures 2b and S5B). Furthermore, H&E staining showed no significant pathological changes in the stomach, intestine, liver, spleen, or kidneys of the BCF-01-treated mice (Figures 2c and S5C).

A. Body weight change in different groups of mice. B. Concentration of ALT, AST, and UREA in the blood. C. Representative picture of HE staining of vital organs.

### BCF-01 inhibits H. pylori infection in vivo

**Figure 3.**
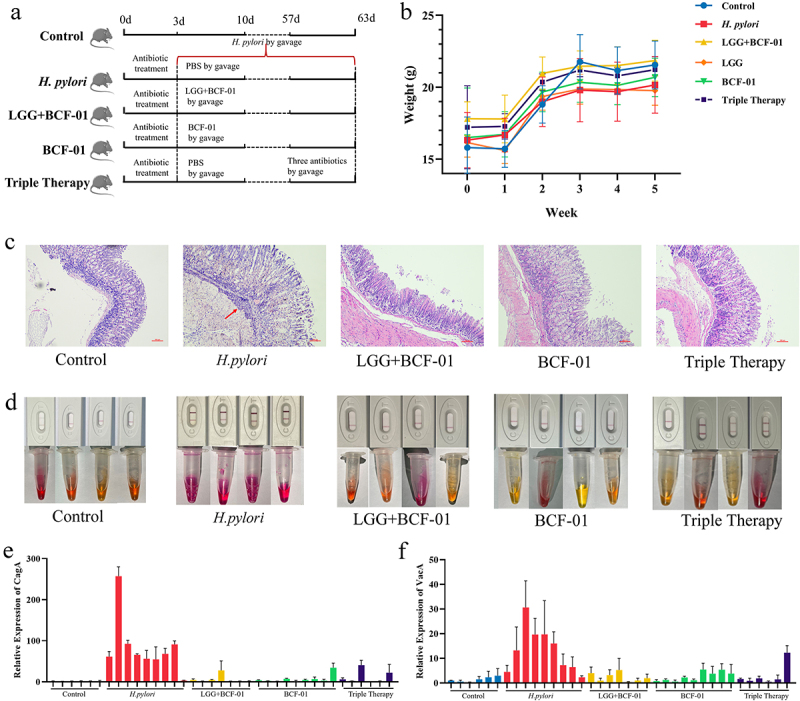
*W. coagulans BCF-01* inhibits Hp infection in a mouse model.

Next, we established an *H. pylori*-infected mouse model and treated these mice with probiotics and triple antibiotic therapy. We implemented probiotic treatment with BCF-01 alone or BCF-01 combined with LGG to assess potential synergistic effects (Figure 3a).

A. Grouping and flowchart of the mouse experiment. B. Body weight change in different groups of mice. C. Representative picture of HE staining of gastro. D. Urease test of gastro contents and fecal Hp antigen detection of feces. E. Transcript levels of CagA in different groups of mice. F. Transcript levels of VacA in different groups of mice.

The growth rate of *H. pylori*-infected mice was generally lower than that of the control group mice; nonetheless, mice treated with probiotics exhibited a higher growth rate than those in the *H. pylori* group (Figure 3b). H&E staining of stomach tissues from mice in each group revealed significant disruptions in the gastric mucosal structure and substantial immune cell infiltration in the *H. pylori* group, whereas gastric tissue structures were better preserved in the BCF-01 and LGG+BCF-01 groups (Figure 3c).

Next, urease and fecal *H. pylori* antigen tests were determined to be positive in the *H. pylori* group, confirming the successful establishment of the *H. pylori*-infected mouse model (Figure 3d). In the BCF-01 and LGG+BCF-01 groups, the gastric mucosa urease and fecal *H. pylori* antigen tests were negative; moreover, these probiotic-treated groups exhibited a higher seroconversion rate than the triple antibiotic therapy group. Interestingly, the LGG+BCF-01 group did not exhibit a significantly higher seroconversion rate than mice treated with BCF-01 alone.

*H. pylori* colonization triggers the production of virulence factors that damage the gastric mucosa, disrupt the mucosal barrier, and induce immune reactions.^24^ Therefore, we investigated whether BCF-01 could reduce *H. pylori* virulence gene expression. The qPCR results revealed significant upregulation of CagA and VacA in the gastric mucosa of the *H. pylori* group, indicating increased gastric colonization and expression of *H. pylori* virulence genes (Figure 3e, f). This *H. pylori* virulence genes CagA and VacA expression in the BCF-01 and LGG+BCF-01 groups were significantly lower than that in the *H. pylori* group, with approaching control group levels (Figure 3e, f).

### BCF-01 restores gastric microbiota homeostasis

Previously, we established that a single dose of BCF-01 was as effective as triple therapy in suppressing *H. pylori* infection; nonetheless, antibiotic treatment often leads to gastrointestinal flora dysbiosis. Therefore, we aimed to determine whether BCF-01 could offer superior improvements in correcting gastric dysbiosis and restoring microbial homeostasis.

**Figure 4.**
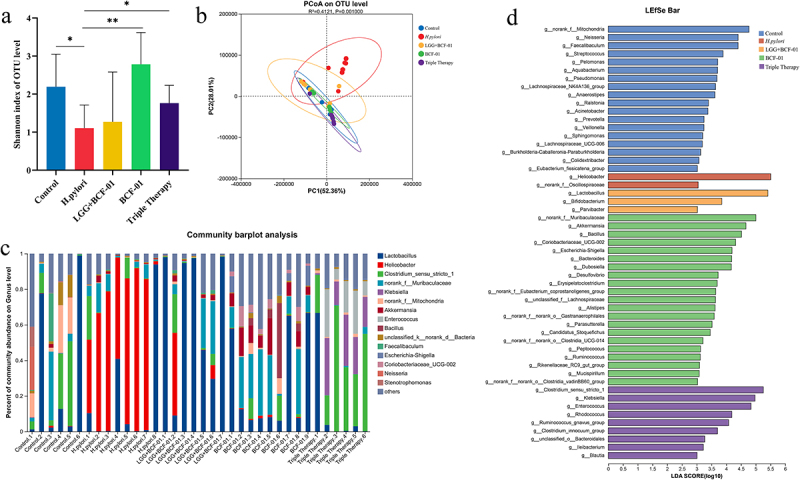
16S rRNA sequencing and bioinformatic analysis of mouse intestinal content.

Specifically, we conducted 16S rRNA sequencing and bioinformatics analysis of the gastric contents from each group. We determined that the α-diversity of the gastric flora in mice was significantly reduced after *H. pylori* infection (Figure 4a). Nonetheless, after BCF-01 intervention, α-diversity significantly increased, showing no statistically significant difference compared with the control group (Figure 4a). Although triple therapy partially improved α-diversity, its effect was inferior to that of BCF-01, with species diversity not fully recovering to the level of the control group (Figure 4a). Principal Coordinate Analysis of β-diversity demonstrated that the microbial composition of the *H. pylori* group formed a distinct cluster (Figure 4b). In contrast, clustering of the BCF-01 and triple therapy groups was similar to that of the control group (Figure 4b). Moreover, we identified *Helicobacter* as the dominant genus in the gastric flora of *H. pylori* group mice; this significantly differed from genera abundances in the other groups (Figure 4c). In the BCF-01 and LGG+BCF-01 groups, gastric *Helicobacter* was mostly eliminated; correspondingly, the abundance of *Bacillus*, *Lactobacillus*, and some potentially beneficial genera (such as *Akkermansia*) was significantly increased (Figure 4c). Although triple therapy also eliminated a large amount of *Helicobacter*, the proportion of potentially pathogenic genera, such as *Enterococcu*s, *Clostridium*, and *Klebsiella*, increased (Figure 4c). Furthermore, LefSe results indicated that *Helicobacter* abundance in the *H. pylori* group was significantly higher than in the other groups (Figure 4d). Conversely, the abundance of *Akkermansia* and other potentially beneficial genera was significantly higher in the BCF-01 group than that in the other groups (Figure 4d). Moreover, *Lactobacillus* was significantly more abundant in the LGG+BCF-01 group than in the other groups. However, the abundance of potentially pathogenic and antibiotic-resistant genera, such as *Enterococcus*, *Klebsiella*, *Staphylococcus*, and *Clostridium*, was significantly higher in the triple therapy group (Figure 4d).

A. Alpha diversity of the microbiome. B. Beta diversity of the microbiome. C. Histogram of the community barplot analysis. D. LefSe analysis of the microbiome. *p < .05; **p < .01.

### BCF-01 modulates mucosal immunity via TLR4-NFκB-pyroptosis signaling inhibition

**Figure 5.**
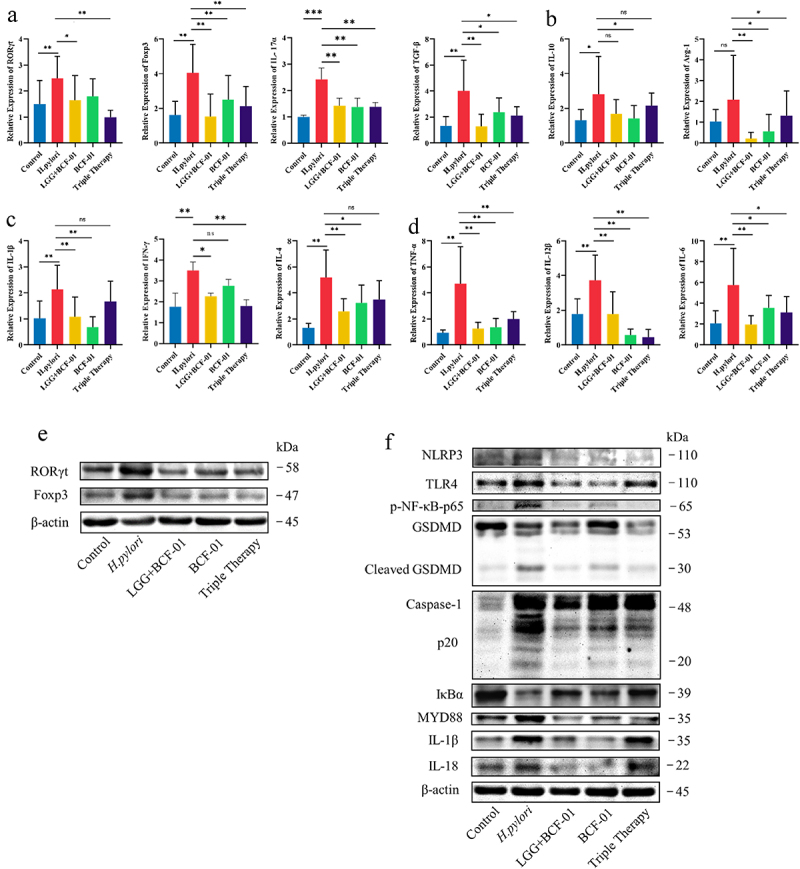
*W. coagulans BCF-01* regulates mucosal immunity through the TLR4-NFκB-pyroptosis signaling pathway.

As human commensal bacteria are involved in regulating the host immune system,^25^ we hypothesized that BCF-01 may also exert its effects by altering the immune status of the host gastric mucosa. Therefore, we validated transcription and protein levels of immune factors in tissues from the different groups of mice. Through qPCR, we detected the key transcription factors of CD4+ T cells, macrophage phenotypic molecules, and classical inflammatory markers. Unlike typical inflammation, the transcript levels of Th1, Th2, Tregs, Th17 cells, pro-inflammatory macrophages, and anti-inflammatory cytokines were upregulated in the *H. pylori* group (Figure 5a–d). Nonetheless, BCF-01 and LGG+BCF-01 restored the immune response induced by *H. pylori*, with their inhibitory effects being comparable to those of triple antibiotic therapy (Figure 5a–d). Finally, we validated classical transcription factor expression in Th17 and Treg cells via western blot analysis. Overall, the abundance of Th17 and Treg cells increased during *H. pylori* infection and was subsequently inhibited by BCF-01 (Figure 5e).

A. Transcript levels of RORγ, Foxp3, IL-17α, and TGF-β in gastro contents B. Transcript levels of IL-10 and Arg-1 in gastro contents C. Transcript levels of IL-1β, IFN-γ, and IL-4 in gastro contents. D. Transcript levels of TNF-α, IL-12, and IL-6 in gastro contents. E. Protein levels of RORγ and Foxp3. F. Protein level of key genes in the TLR4-NFκB-pyroptosis signaling pathway. *p < .05; **p < .01; ***p < .001.

The TLR4-NFκB pathway is a classic pathway associated with *H. pylori* pathogenesis; moreover, previous bioinformatic and experimental evidence has suggested that pyroptosis may be involved in *H. pylori* infection.^26^ Western blotting of TLR4-NFκB pathway – associated proteins indicated that *H. pylori* significantly activated these proteins, leading to the activation of Caspase-1 and GSDMD, release of IL-1β and IL-18, and induction of cell pyroptosis (Figure 5f). BCF-01 intervention significantly reduced TLR4-NFκB and pyroptosis activation, with its inhibitory effects being superior to that of triple therapy (Figure 5f).

We also evaluated gastric barrier – related proteins and determined that *H. pylori* infection downregulated tight junction protein expression, whereas BCF-01 effectively increased their expression (Figure S6). Furthermore, LGG+BCF-01 enhanced tight junction protein expression more than BCF-01 alone, suggesting that the combined use of multiple probiotics protects the gastric mucosal barrier (Figure S6).

**Figure 6.**
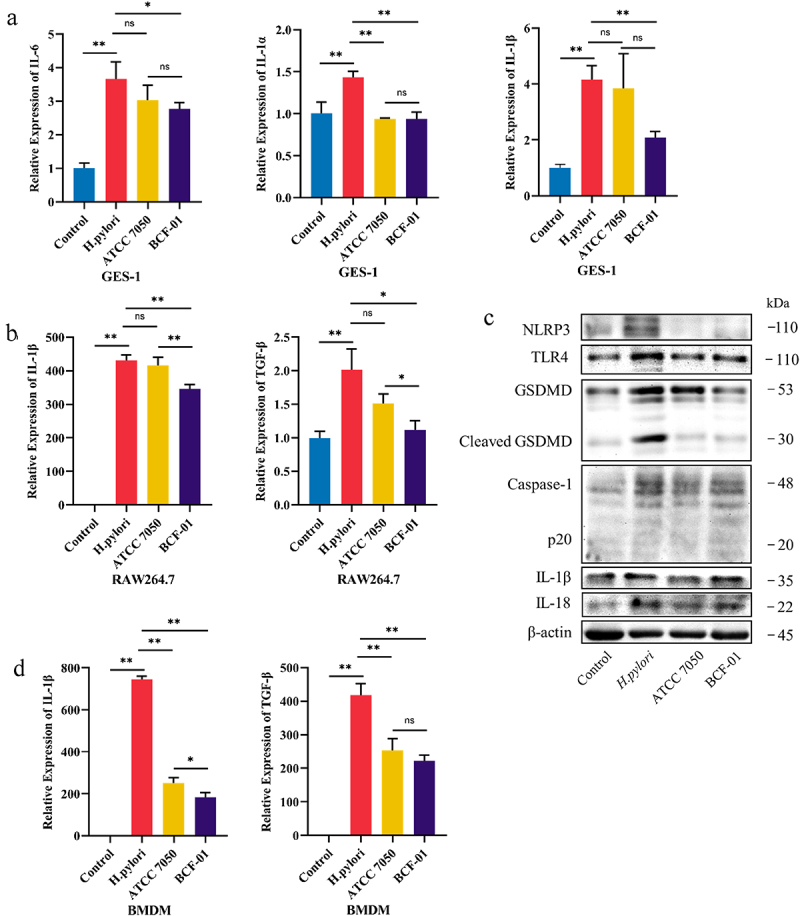
*W*. *coagulans* BCF-01 downregulates the TLR4-NFκB-pyroptosis signaling pathway in macrophages.

Finally, we conducted *in-vitro* mechanistic validation analysis. We used GES-1 cells to simulate the gastric mucosal structure, which was treated with *H. pylori* to establish a cell infection model; BCF-01 was administered as a preventive treatment. Corresponding qPCR results demonstrated that after *H. pylori* infection, GES-1 cells exhibited elevated transcription levels of inflammatory cytokines, such as IL-6, IL-1α, and IL-1β; conversely, the expression of tight junction proteins ZO-1 and Occludin was downregulated (Figures 6A and S7). However, pretreatment with BCF-01 led to reduced transcription levels of these inflammatory cytokines and increased levels of tight junction protein (Figures 6a and S7).

A. Transcript levels of IL-6, IL-1α, and IL-1β in GES-1 cells. B. Transcript levels of IL-1β and TGF-β in RAW264.7 cells. C. Protein levels of key genes in the TLR4-NFκB- pyroptosis signaling pathway in RAW264.7 cells. D. Transcript levels of IL-1β and TGF-β in BMDM cells. *p < .05; **p < .01; ***p < .001.

Since macrophages are important target cells for *H. pylori* infection,^27^ we established an *H. pylori* infection model in RAW264.7 cells. This *H. pylori* infection increased the secretion of the classical inflammatory cytokines IL-1β and TGF-β from macrophages (Figure 6b). Furthermore, western blot analysis of the TLR4-NFκB-pyroptosis pathway indicated signaling pathway activation following *H. pylori* infection; ultimately, this resulted in the upregulation of the downstream proteins Caspase-1, GSDMD, IL-1β, and IL-18 (Figure 6c). Nonetheless, BCF-01 treatment resulted in significant downregulation of this activated pathway (Figure 6c). Finally, primary macrophages derived from mouse bone marrow were used to better simulate *H. pylori* infection. The qPCR validation reaffirmed that *H. pylori* infection increased secretion of IL-1β and TGF-β from macrophages, while preventive treatment with BCF-01 inhibited macrophage activation (Figure 6d).

## Discussion

Prior studies have explored the use of probiotics as a therapeutic strategy for *H. pylori* infection, with a predominant focus on the classical probiotic *Lactobacillus*; however, understanding regarding the corresponding impact and application of *W. coagulans* remains limited.^28^ *W. coagulans* exhibits antibacterial effects, influencing *H. pylori* cell proliferation and survival through various mechanisms. These include the production of beneficial substances, such as organic acids and antimicrobial peptides, competition with *H. pylori* for host cell adhesion receptor binding, and modulation of host immune responses.^29^ *W. coagulans* also regulates gastrointestinal microbiota homeostasis by enhancing the abundance of beneficial bacteria while suppressing harmful bacteria.^30–32^ This improved homeostasis may reduce the risk of *H. pylori* infection and related diseases. Treatment of *H. pylori* infection typically involves antibiotic combination therapy; however, given the increasing resistance of *H. pylori* to common antibiotics, alternative treatments have become increasingly important.^33^ *W. coagulans* holds promise as a standalone or adjunctive therapy with traditional antibiotics. Clinical studies have determined that *W. coagulans* can effectively inhibit *H. pylori* with fewer adverse events, thereby alleviating the burden of antibiotic resistance.^34^

*W. coagulans* is a widely studied bacterial genus isolated from various sources, including soil, plants, and food.^35–38^ Moreover, some strains have been isolated from the human gastrointestinal tract, including *W. coagulans* MTCC 5856 and SNZ1969.^39,40^ However, isolation of *W. coagulans* from the human stomach has not been previously reported. In the present study, we successfully isolated five novel *W. coagulans* strains (BCF-01–05) from human gastric mucosal tissues. Further, we demonstrated that these strains, especially BCF-01, exhibited superior acid resistance, gastric colonization capability, and anti- *H. pylori* activity compared to ATCC strains. This highlights the potential clinical application of these *W. coagulans* strains, especially BCF-01, as probiotics for *H. pylori* infection management.

Our *in-vitro* and *in-vivo* experiments revealed the close association between *W. coagulans* BCF-01 and mucosal immunity post- *H. pylori* infection. Specifically, it strengthens the gastric mucosal barrier, suppresses TLR4-NFκB-pyroptosis signaling, inhibits the differentiation of pro-inflammatory macrophages and pro-inflammatory CD4+ T cells, significantly inhibits *H. pylori* colonization, and accelerates the establishment of the gastric microbial community. These findings align with previous reports of *W. coagulans* strains colonizing the intestinal mucosa, whereby they enhance mucosal barrier function, modulate the mucosal immune system, reduce inflammatory factor production, alleviate intestinal inflammation and inflammation-related diseases, promote secretory IgA production, and facilitate antigen-specific immune responses.^41^

There were four limitations to this study. Firstly, only one gender of mice was used in the infectious model of *H. pylori*. Secondly, the precise molecular mechanisms underlying BCF-01-mediated regulation of macrophages *in vivo* remain elusive. Subsequently, the effects of BCF-01 on other immune cell populations, including CD4 cells, have not been thoroughly investigated. Lastly, the dearth of validation using a larger cohort of clinical specimens is a notable limitation.

**Figure 7.**
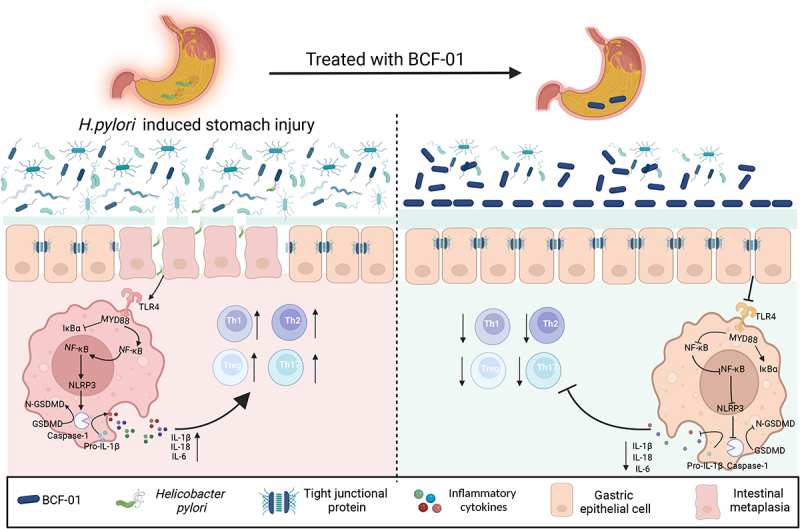
Schematic diagram summarizing the working model used in this study.

In conclusion, we successfully isolated five novel *W. coagulans* strains (BCF-01–05) from human gastric mucosal tissue and demonstrated their superior acid resistance, gastric colonization capability, and anti-*H. pylori* activity. Specifically, BCF-01 effectively restored gastric microecology, improved *H. pylori*-mediated mucosal barrier destruction, and reduced inflammation via inhibition of TLR4-NFκB-pyroptosis signaling in macrophages (Figure 7). Overall, these *W. coagulans* strains hold promise as potential alternatives to traditional triple therapy for *H. pylori* infection prevention, offering minimal side effects.

## Supplementary Material

suppl figure.docx

## Data Availability

The data that support the findings of this study have been deposited in the CNGB Sequence Archive (CNSA) of the China National GeneBank DataBase (CNGBdb) under accession number CNP0005043.
