# Supplementary material for: Weizmannia coagulans BCF-01: a novel gastrogenic probiotic for Helicobacter pylori infection control
Source: Gut Microbes. 2024 Feb 9;16(1):2313770. doi: 10.1080/19490976.2024.2313770 (PMC10860349; doi:10.1080/19490976.2024.2313770)
Supplement: suppl figure.docx [file KGMI_A_2313770_SM0865.docx]

**
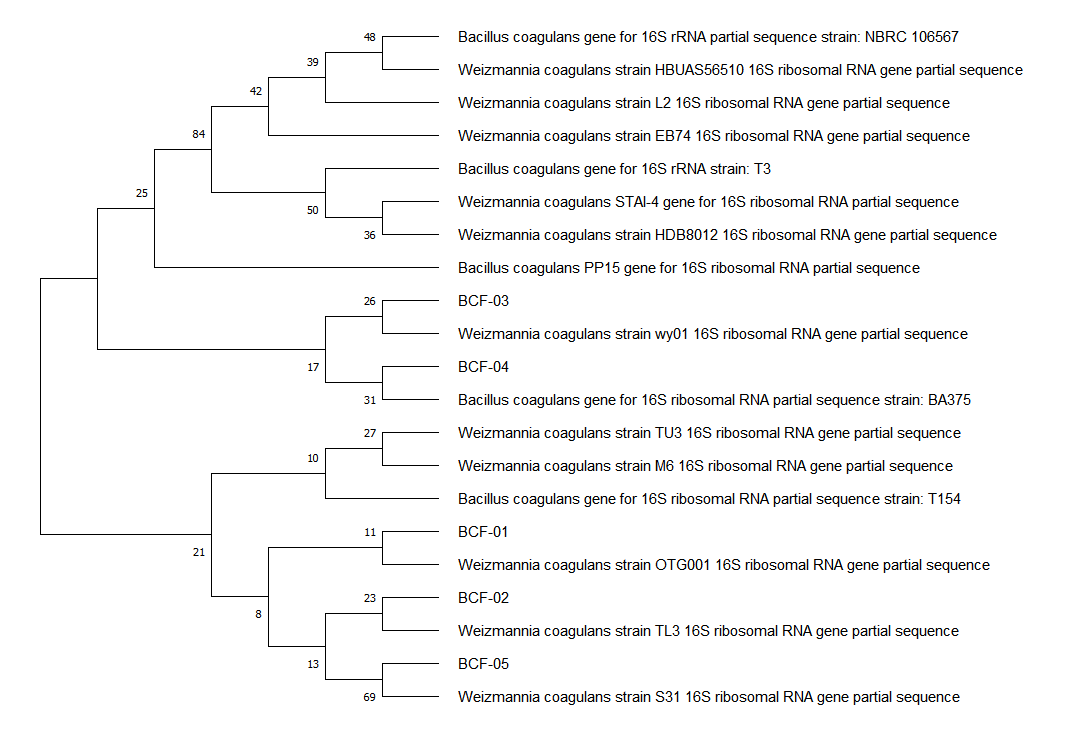
**

**Supplementary Fig. 1 The** **phylogenetic tree of 5 novel *W. coagulans* strains.**

**
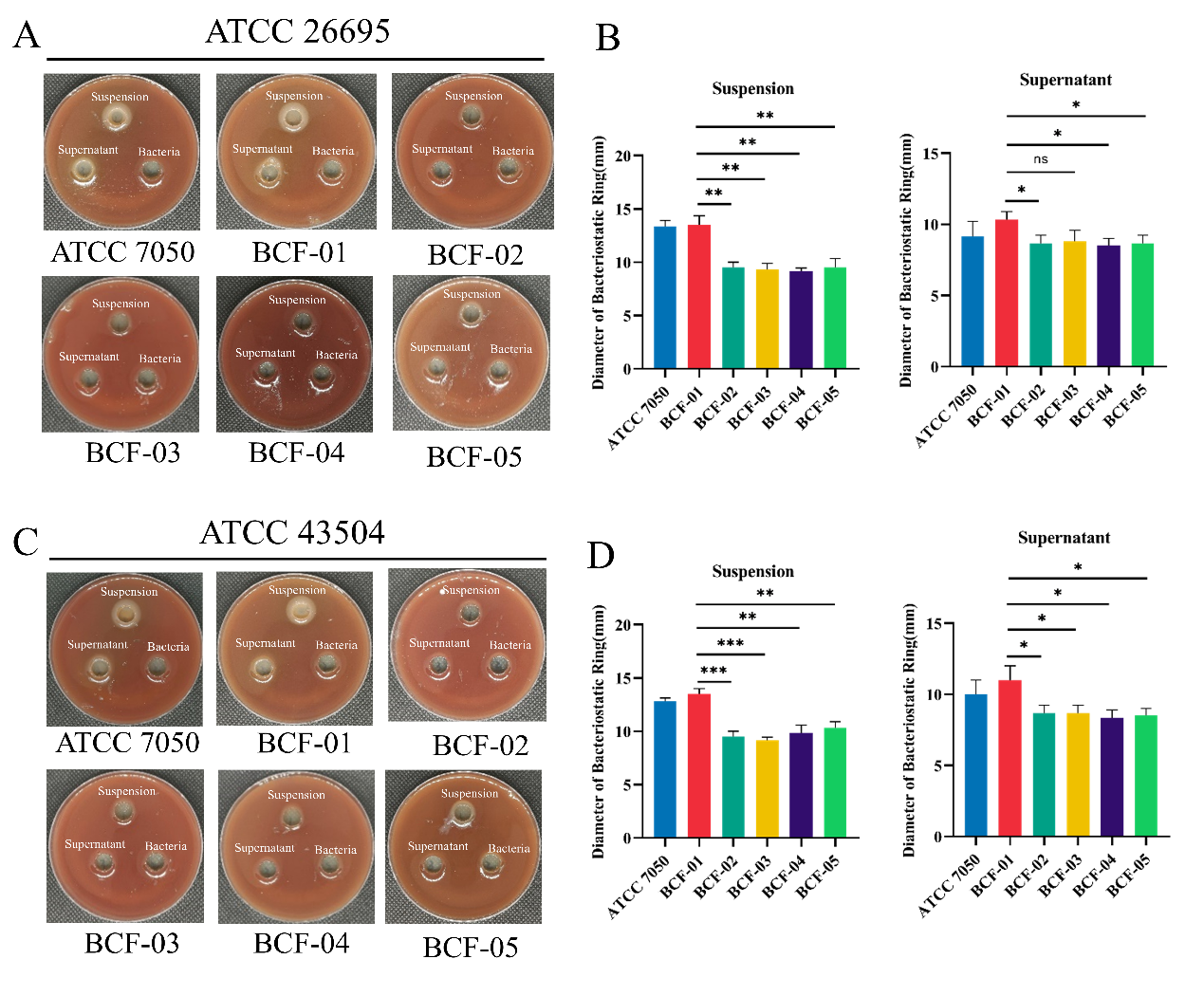
**

**Supplementary Fig. 2 The antibacteria test of 5 novel *W. coagulans* strains and *H.polyri*.** A. Representative picture of antibacteria test of *W. coagulans* strains and ATCC 26695. B. Diameter of inhibition zones of suspension and supernatant. C. Representative picture of antibacteria test of *W. coagulans* strains and ATCC 43504. D. Diameter of inhibition zones of suspension and supernatant. *P<0.05; **P<0.01; ***P<0.001.


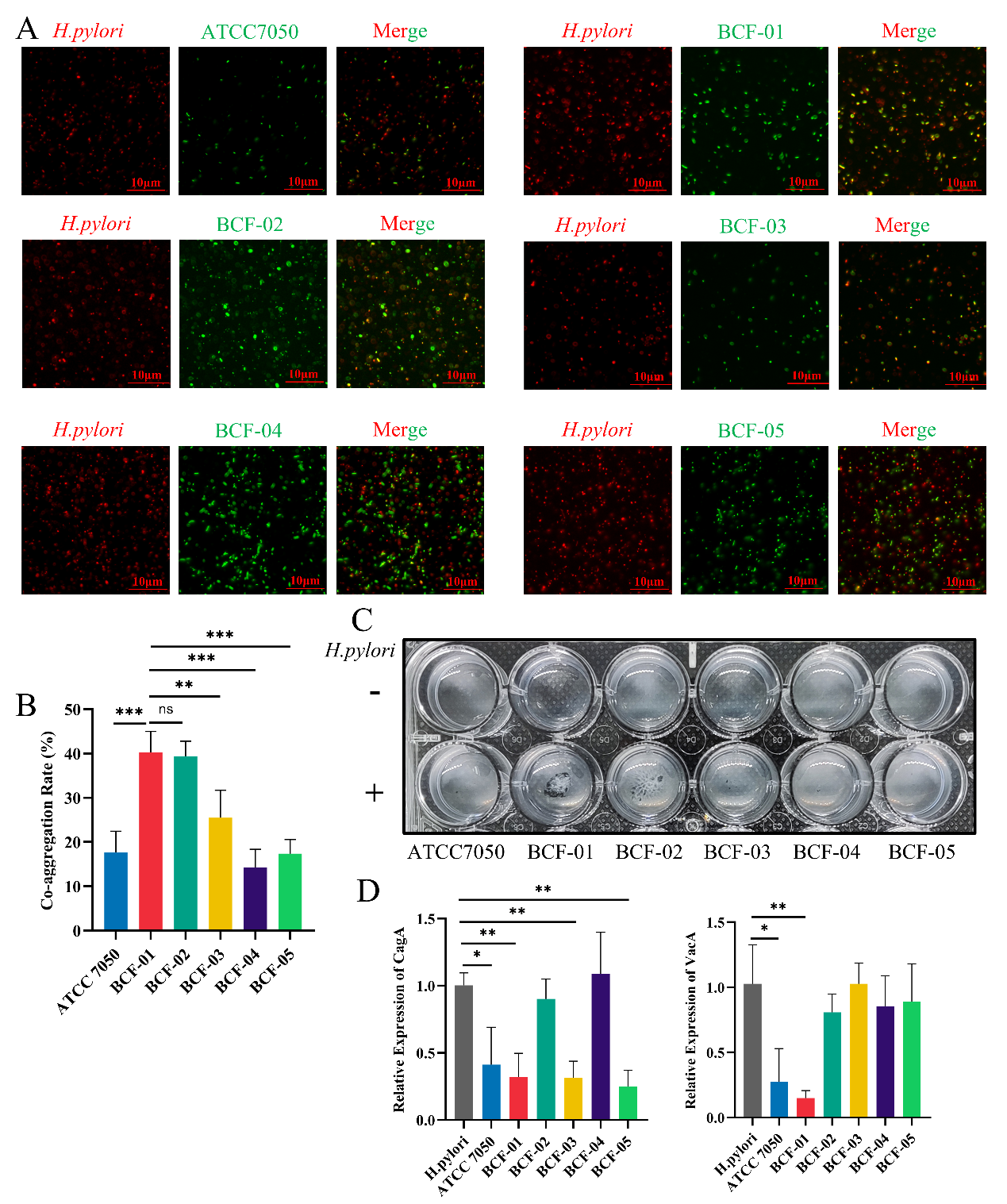


**Supplementary Fig. 3 Microscopic analysis of co-aggregation of *W. coagulans* strains and *H.polyri* SS1 in artificial stomach juice (pH 4).** A. *H. pylori* SS1 stained with

HI and *W. coagulans* strains stained with CFDA. B. The co-aggregation rate of *W. coagulans* strains and *H.polyri* SS1. C. Co-aggregation of *W. coagulans* strains and *H.polyri* SS1 is macroscopically visible. D. The relative abundance of CagA and VacA of *H.polyri* SS1 after incubation with *W. coagulans* strains. *P<0.05; **P<0.01; ***P<0.001.

**
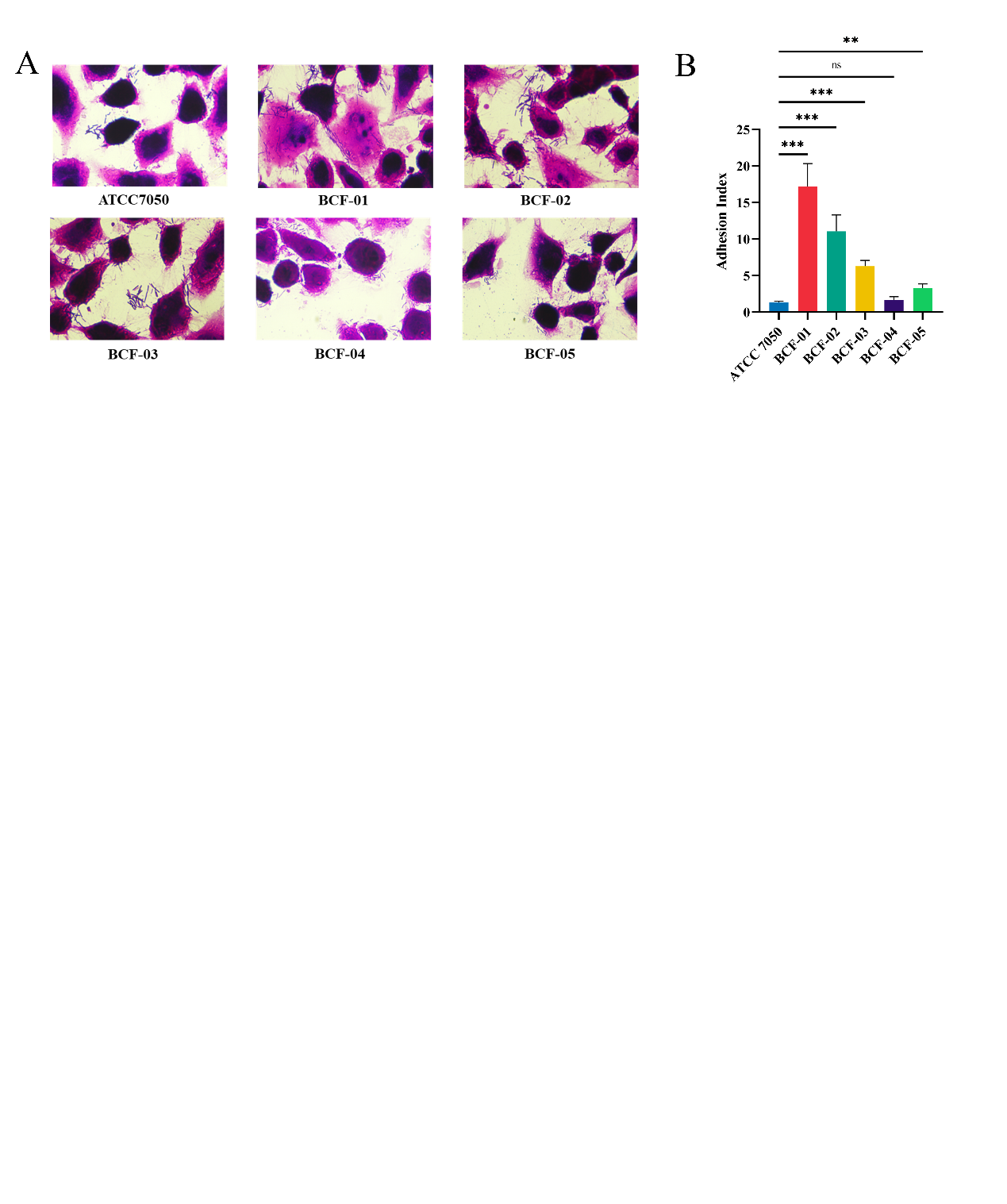
**

**Supplementary Fig. 4 The adhersion of 5 novel *W. coagulans* strains to GES-1 cells through staining counting method.** A. Representative picture of *W. coagulans* adhering to GES-1 cells. B. Adhesion rate of different *W. coagulans*. *P<0.05; **P<0.01; ***P<0.001.


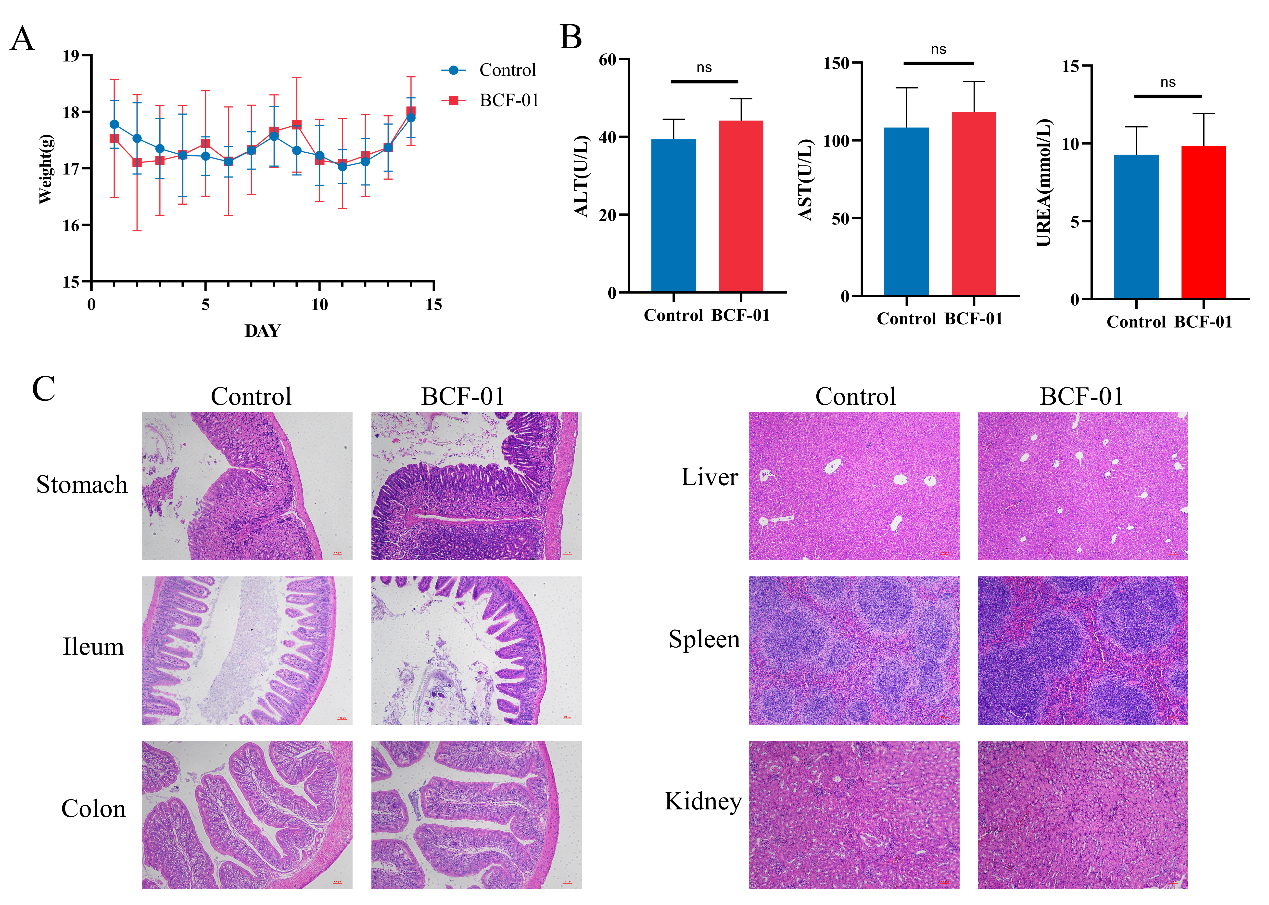


**Supplementary Fig. 5 Safety evaluation of gastrogenic *W. coagulans* *BCF-01* in female mice.** A. Body weight change of different groups of mice. B. The concentration of ALT, AST and UREA in the blood. C. Representative picture of HE staining of vital organs.


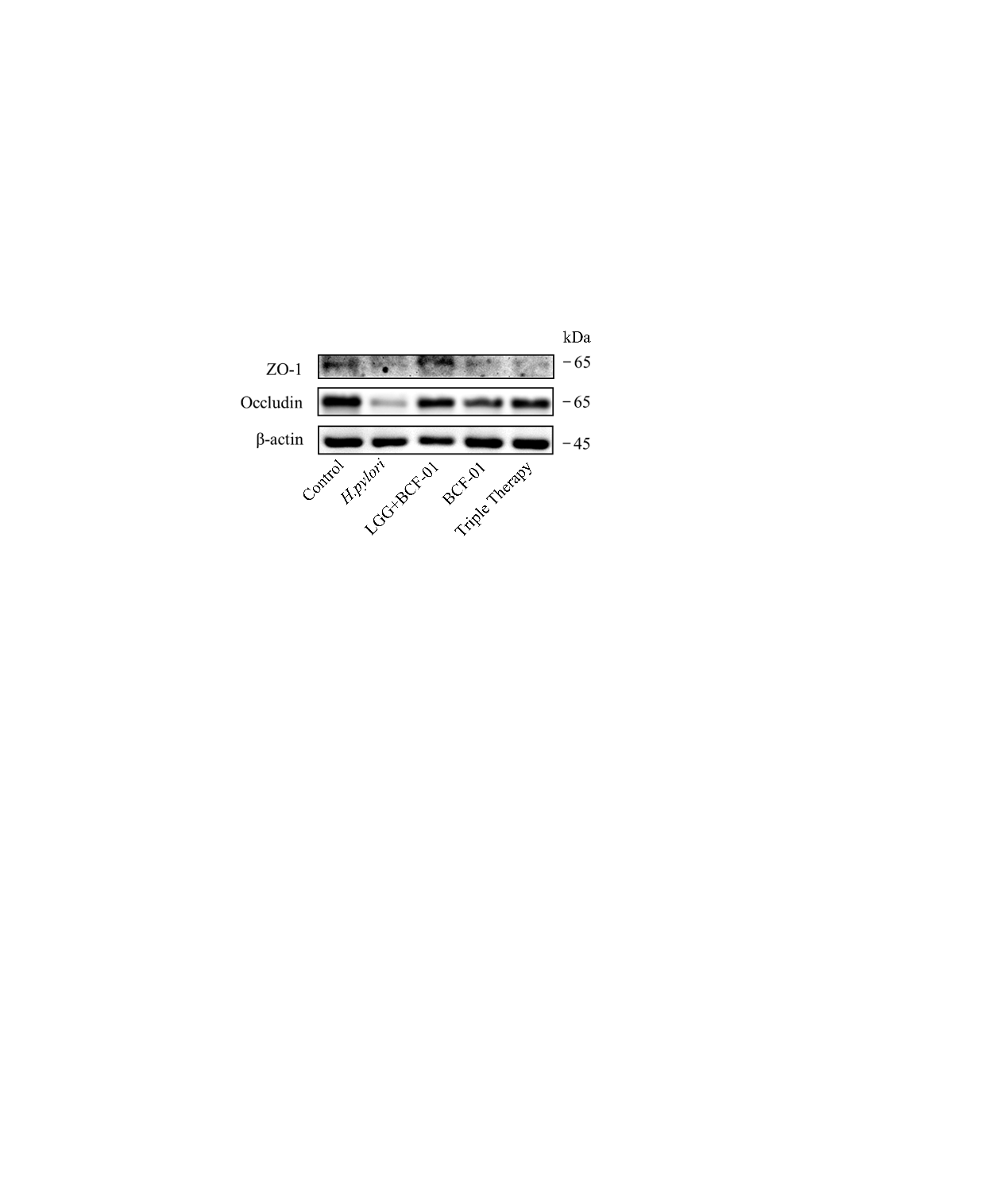


**Supplementary Fig. 6** **Protein level of tight junction proteins in mouse gastro.**


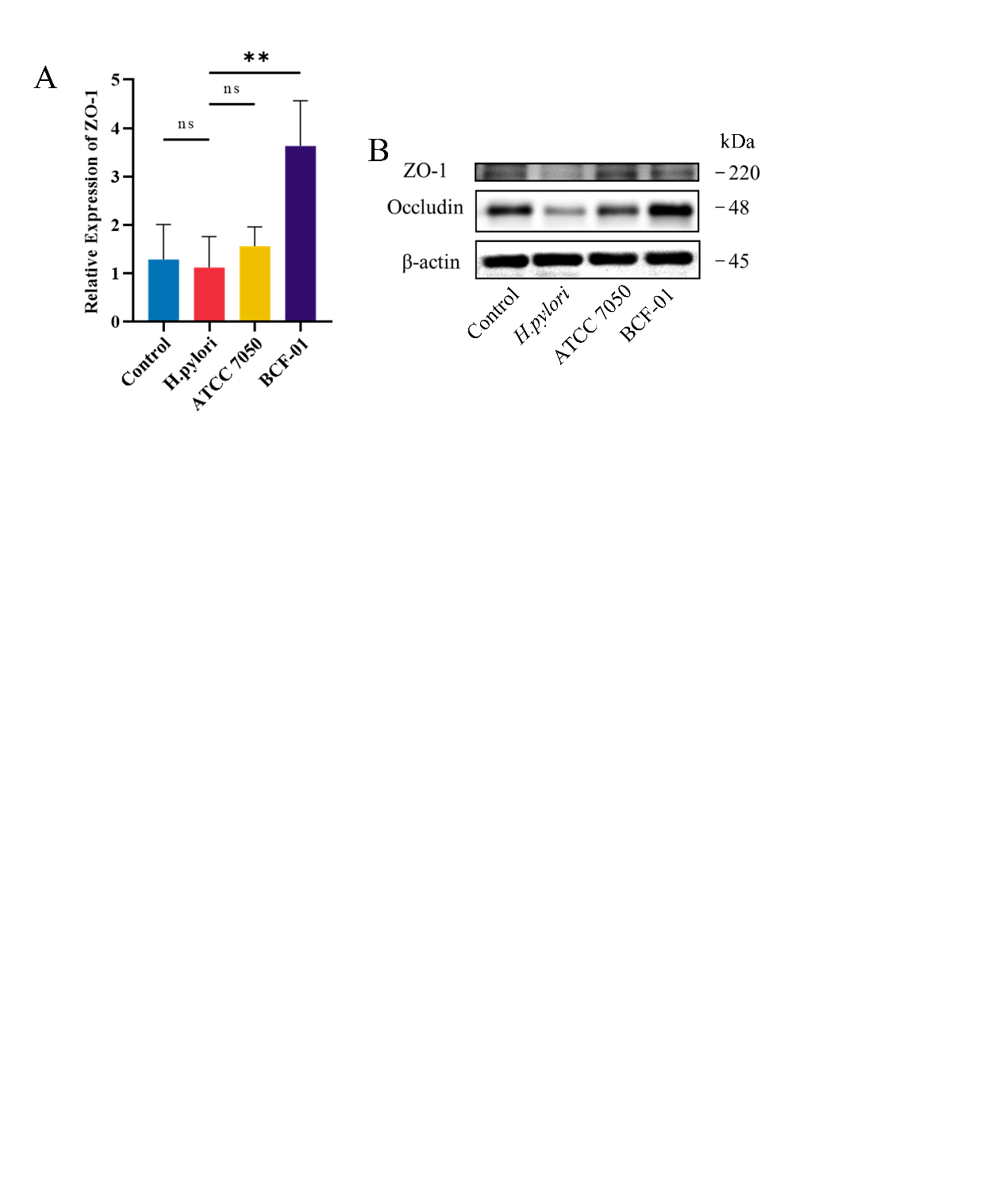


**Supplementary Fig. 7 BCF-01 upregulated the tight junction proteins in GES-1 cells.** A. Transcript level of ZO-1. B. Protein level of ZO-1 and occludin. *P<0.05; **P<0.01.
